# Spinal mobility and muscle function in middle-aged patients treated for early onset idiopathic scoliosis: compared with untreated and treated adolescent onset patients

**DOI:** 10.1007/s43390-022-00487-8

**Published:** 2022-03-23

**Authors:** Karin Romberg, Aina Danielsson, Monika Fagevik Olsén, Gunilla Kjellby-Wendt

**Affiliations:** 1 Department of Health and Rehabilitation, Institute of Neuroscience and Physiology, Sahlgrenska Academy, University of Gothenburg, Box 455, 405 30 Gothenburg, Sweden; 2 Department of Physical Therapy, Sahlgrenska University Hospital, 413 45 Gothenburg, SE Sweden; 3 Department of Orthopaedics, Institute of Clinical Sciences, Sahlgrenska Academy, University of Gothenburg, Gothenburg, Sweden; 4 Department of Orthopaedics, Sahlgrenska University Hospital, Gothenburg, Sweden

**Keywords:** Idiopathic scoliosis, Spinal mobility, Muscle function, Long term

## Abstract

**Purpose:**

To determine long-term outcome in terms of spinal range of motion (ROM) and trunk muscle endurance (TME) patients treated for idiopathic scoliosis, diagnosed before the age of ten, were evaluated and compared with untreated or treated patients with idiopathic scoliosis with adolescent onset (AIS).

**Methods:**

Sixty-three braced and 53 operated patients underwent examination of spinal ROM and TME. Validated questionnaires were used for evaluation of back function.

**Results:**

A total of 116 patients were examined 26.5 years after treatment. Braced EOS patients had longer bracing time and operated EOS patients had longer fusions compared to the respective AIS groups. Braced EOS patients had similar total ROM (thoracic ROM 40°, lumbar ROM 78°) and TME (trunk flexors 140 s, trunk extensors 255 s) as untreated AIS patients (thoracic ROM 34°, lumbar ROM 88°, trunk flexor endurance 158 s, trunk extensor endurance 234 s). Braced patients also had significantly better results than braced AIS patients. Operated EOS patients were slightly but significantly stronger and more mobile compared to AIS peers. The lumbar ROM was found to affect the back function in the operated EOS group (Oswestry Questionnaire, rs = 0.49, *p* < 0.001).

**Conclusions:**

The braced EOS patients had mostly similar muscle strength and mobility as the untreated but younger AIS group, while the braced AIS group showed reductions of both strength and mobility. Similar significant, but small, differences were also found between operated EOS and AIS patients. Especially for muscle strength were findings at a level that would be of significant clinical importance.

**Levels of evidence:**

III.

## Introduction

Idiopathic scoliosis is defined as early onset when the debut age is before the age of ten [1]. Compared to the adolescent idiopathic scoliosis (AIS), which debuts after the age of ten, there is an increased risk in the early onset scoliosis (EOS) for an end-result with larger curve magnitude, greater kyphosis and a more distal curve apex [2]. The curve is more likely to progress, especially during the prepubertal growth period [3, 4], less likely to respond to bracing and more likely to require surgical treatment [5]. The EOS patients are, therefore, often treated with a full-time brace for several years or with a spinal fusion.

The natural history of untreated AIS does not result in increased mortality or severe long-term health issues though back pain is more frequent [6, 7]. Patients with untreated EOS on the other hand, run an increased risk for respiratory failure and premature death [8].

Treatment of EOS is aimed to alter the outcome so that respiratory problems will be prevented and the life span becomes close to normal. From that perspective, other aspects of outcome become more important, such as pain or function in daily life. Impaired function of trunk muscle flexors and extensors have been shown to be closely associated to the pathogenesis of chronic low back pain [9–13].

Back pain has been shown to be frequent among adult individuals with childhood scoliosis, even if the effect on quality of life and daily function is not very large. In a previous long-term follow-up of 237 patients with adolescent onset of idiopathic scoliosis (IS), it was found that both brace treated and surgically treated patients had reduced spinal range of motion (ROM) and reduced trunk muscle endurance (TME) compared to healthy controls [14]. For the surgically treated patients, it was found that higher trunk extensor and flexor muscle endurance or a better lumbar spinal mobility correlated with better physical function measures (Oswestry Disability Index) [14].

In two other studies, patients with IS with either early, or adolescent, onset treated with a Boston brace, were shown to have similar and satisfactory long-term results to one another in terms of curve progression and health related quality of life [15, 16]. The status of spinal ROM and TME was, however, not reported in those studies.

We have not found any reports on spinal ROM or TME in patients with early onset of IS, neither on braced nor on operated patients. These individuals may run the risk of being even more affected than those with adolescent onset due to their earlier onset which often results in larger deformities and a prolonged brace treatment period.

The aim of this long-term follow-up study was to evaluate the spinal mobility, trunk muscle endurance and stabilizing trunk muscle function in adult patients treated for early onset scoliosis, with brace or surgery, during childhood or adolescence. The aim was also to explore correlations of spinal ROM, TME with back pain, function and physical activity. Further aims were to compare these results with adult patients adolescent onset of scoliosis that were treated before maturity and to explore existing differences towards untreated AIS patients.

## Methods

### Patients

**Fig. 1:**
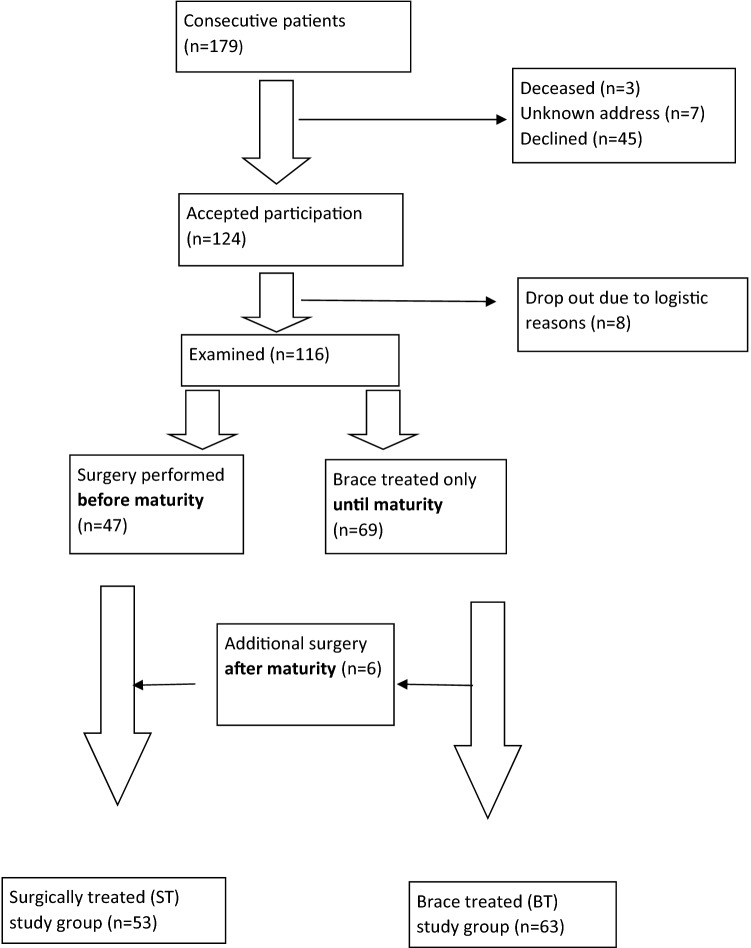
Flowchart of included patients

The Gothenburg Scoliosis Databank, which contains consecutive information about all patients with scoliosis at the Department of Orthopaedics at Sahlgrenska University Hospital, Gothenburg, Sweden, between 1966 and 1994, was used to identify the study population. Patients included had (1) a diagnosis of IS before the age of 10, (2) treatment with either a brace (BT, for at least 6 months) or with surgery (ST), (3) no other related disorders of spine anomalies and (4) > 10 years since skeletal maturity or surgical procedure. The original series consisted of 179 consecutive patients, of which 116 completed this study (Fig. 1). The mean age at diagnosis was 7.0 years (0.1–9.8), but only 9 (8%) of the individuals were diagnosed before the age of four years, so the group mainly consists of juvenile onset patients. Treatment started between 1966 and 1992. Sixty-three patients were braced solely until skeletal maturity, with a Milwaukee brace until 1974 (in 26 patients) and thereafter with a Boston brace (in 37 patients). All together 53 patients were surgically treated before skeletal maturity, of whom 33 were initially braced but operated before skeletal maturity due to curve progression. A further six patients (out of the 53) were braced until maturity but later operated, all before the age of 22 years, due to a significant curve size at skeletal maturity. The surgical procedure was performed with Harrington instrumentation until 1995 (in 50 patients) and thereafter with the Isola system (in 3 patients).

Unpublished data collected during two previously performed long-term follow-up studies with focus on patients with idiopathic scoliosis with adolescent onset, i.e., after the age of ten years, were used for comparison of the results:

1. Patients with AIS, treated between 1968 and 1977 at our department, with the same inclusion criteria as the “EOS”-group except for age of diagnosis and the same treatment regimen. [14]. Out of a consecutive series of 283 patients, 102 braced and 135 surgically treated patients were examined.
2. Untreated patients with AIS: forty out of 65 originally untreated patients with AIS, that had solely been observed at our department until skeletal maturity as the intention to treat, were examined [17]. Patients were recruited during adolescence [18] with the following criteria: (1) diagnosis of idiopathic scoliosis after the age of 10, (2) a thoracic or thoracolumbar curve of moderate size (25°–35°).

### Description of the curve

A full standing posterior–anterior digital roentgenogram was performed at the follow-up in all patients and curve size was measured using the Cobb method [19]. All patients underwent measurement of trunk deformity by use of a Bunnell scoliometer [20].

### Examination of spinal range of motion and trunk muscle endurance

The present study group was examined at the follow-up by exactly the same methods, all validated and extensively used, as in the previous studies. They were all performed by the same physiotherapist (the first author).

Patients were evaluated for spinal range of motion [21–29] and evaluation of trunk muscle endurance [30], as explained in detail in Tables 1 and 2. Cervical range of motion in flexion, extension, lateral bending and rotation was evaluated in a sitting position with a Myrin inclinometer [24].

**Table 1:**
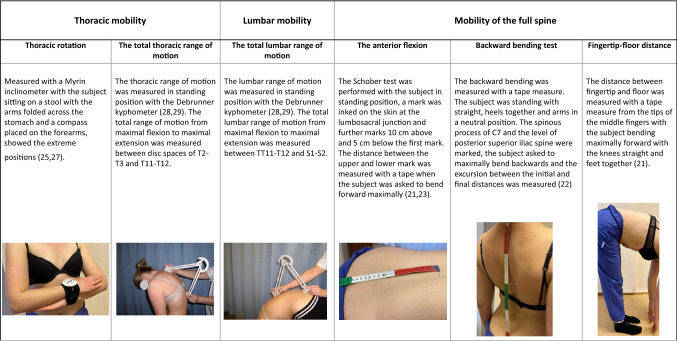
Methods for evaluation of spinal range of motion

### Questionnaires concerning back pain and function

The Oswestry Low Back Pain Disability Questionnaire (ODI) was used as disease-specific questionnaire for evaluation of the general back function [31]. For information about the level of physical activity during work and leisure time questions from a WHO questionnaire were used [32], Table 3. A visual analog scale (0–100) was used for quantification of the subjective feeling of back stiffness [33], with zero being no feeling of stiffness and 100 the worst possible stiffness.

**Table 2:**
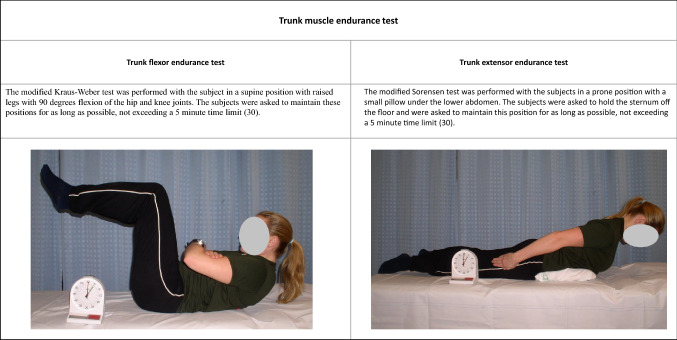
Methods for evaluation of trunk muscle endurance

### Statistical methods

Distributions of variables are given as means, standard deviations and 95% confidence interval (CI). For comparison of continuous variables between two groups the Mann–Whitney *U* test was used and between the three groups the Kruskal Wallis test.

Spearman rank correlation coefficient was used for correlation analysis. Correlation was defined as: little, if any (rs < 0.25), low (rs = 0.26–0.49), moderate (rs = 0.50–0.69), high (rs = 0.70–0.89) and very high (rs = 0.9–1.00) [34].

All significance tests were two tailed, and were conducted at the 5% significance level.

## Results

### Description of demographic data and of the scoliotic curve

Table 4 depicts basic information about all the study groups. The majority of the patients with early onset scoliosis were female. Mean age at start of treatment was 10.6 years for the BT and 13.2 years for the ST patients (*p* < 0.001), and age at the follow-up was 40.8 and 42.4 years, respectively (*p* = 0.187). The mean duration of the brace treatment was 4.8 years, with a maximum length of 11 years. Curve size was similar at the present follow-up (BT 34.7° and ST 36.8°, *p* = 0.381). The length of the fusion had a mean of 10.6 vertebrae (range 5–17).

**Table 3:** Questionnaires used in the follow-up of all the three groups of patients

Questionnaire	Purpose	Description
Oswestry Low Back Pain Disability Questionnaire [31]	Disease-specific QL; general back function	Answers to 10 questions on activities that are back-dependent are scored and summarized. The scoring goes from 0 to 100, where 0 is the least possible disability
WHO questionnaire [32]	Physical activity during work and leisure time	Physical activity during work: 1 = Sedentary 2 = Light work with some physical activity 3 = Relatively heavy work 4 = Heavy manual work Physical activity during leisure time: 1 = Mainly sedentary 2 = Light exercises and training minimum of 4 h/week 3 = Regular training and exercise 4 = Serious training and competitive sports

Sixty-three patients with early onset (BT = 30, ST = 33) did not attend for follow-up and did not differ from the examined group of patients in terms of gender and curve size at start and end of treatment.

The patients with an earlier onset of their scoliosis had started their treatment period significantly earlier compared to those with adolescent onset, at a mean age of 10.6 vs. 14.4 for braced and 13.2 vs. 15.0 for operated, and at a smaller curve size, 28.5° vs. 32.9° for braced and 57.1° vs. 62.1° for operated. The length of the bracing period was significantly longer for those with earlier onset, mean 4.8 vs. 2.7 years. At follow-up, curve size was similar in all the groups, regardless if treated or not, with mean values between 34.7° and 37.7°, n.s. All the treated patients, regardless of their onset of scoliosis, were around 40 years of age, while the untreated patients had a mean age of 32 years (Table 4).

### Spinal range of motion

#### Cervical range of motion

Comparisons were made between the braced and operated EOS patients, and between the two EOS groups and their respective AIS groups. Statistically significant differences were found in only one direction out of the six tested for each comparison, but none were above a mean of 7°. This was also found for the braced EOS patients versus the untreated AIS patients. The operated EOS patients had larger reductions of ROM (by mean 3.7°–9.3°) when compared to the untreated AIS patients.

Cervical flexion in operated EOS patients with fusion from T4 or above (*n* = 30) and from T5/below (*n* = 16) did not differ from similar AIS patients.

#### Thoracic and lumbar range of motion

Results from spinal mobility examinations are reported in Table 5.

**Table 4:** Demographic data/the scoliotic curve in patients with idiopathic scoliosis, depending on treatment type and age of onset. Mean (SD), median [CI 95%: lower, upper.] or n (%)

	Brace-treated patients			Surgically treated patients				Untreated patients
	Onset until age 10 n = 63	Adolescent onset n = 102	p-value	Onset until age 10 n = 53	Adolescent onset n = 135	p-value	p-value Onset until age 10 BT vs ST	Adolescent onset n = 40
Demographic data								
Gender female	54 (86%)**	98 (96%)	0.017	42 (79%)***	125 (93%)	0.009	0.360	40 (100%)
Age at start of treatment (y)	10.6 (2.8) 10.7 [9.91, 11.3]	14.4 (1.4) 14.4 [14.1, 14.7]	< 0.001	13.2 (2.1) 13.3 [12.6, 13.8]	15.0 (1.8) 15.0 [14.7, 15.3]	< 0.001	< 0.001	-
Duration of brace treatment (y)	4.8 (2.6) 4.4 [4.16, 5.44]	2.7 (1.0) 2.5 [2.51, 2.89]	< 0.001	-	-	-	-	-
Age at surgery (y)	-	-	-	13.2 (2.1) 13.3 [12.6, 13.8]	15.0 (1.8) 15.0 [14.7, 15.3]	< 0.001		
Age at present study (y)	40.8 (6.6) *** 40.2 [39.2, 42.4]	39.2 (2.3) *** 39.3 [38.8, 39.6]	0.04	42.4 (7.3) *** 42.9 [40.4, 44.4]	39.6 (2.4) *** 39.7 [39.2, 40]	0.007	0.187	31.8 (1.4) 32.0 [31.4, 32.2]
Follow-up time (y) from completed treatment until present follow-up	24.8 (6.1) *** 24.9 [23.3, 26.3]	22.1 (1.9) *** 21.9 [21.7, 22.5]	0.001	28.5 (7.9) *** 30.0 [26.4, 30.6]	23.2 (1.5) *** 23.0 [22.9, 23.5]	< 0.001	0.006	15.6 (1.4) 15.5 [15.2, 16]
The scoliotic curve								
Curve size								
at start of treatment, degrees	28.5 (8.5) 30.0 [26.4, 30.6]	32.9 (9.5) * 33.0 [31.1, 34.7]	0.008	57.1 (13.1) *** 55.0 [53.6, 60.6]	62.1 (13.3) *** 58.0 [59.9, 64.3]	0.02	< 0.001	29.6 (4.0) 28.0 [28.4, 30.8]
at end of treatment, degrees	23.1 (11.2) *** 23.5 [20.3, 25.9]	29.7 (11.0) 33.0 [27.6, 31.8]	0.001	28.7 (12.1) * 26.0 [25.4, 32]	33.1 (9.5) 34.0 [31.5, 34.7]	0.002	0.056	31.1 (6.0) 30.5 [29.2, 33]
at present follow-up, degrees	34.7 (16.9) 34.0 [30.5, 38.9]	37.7 (14.2) 36.5 [34.9, 40.5]	0.126	36.8 (13.7) 35.0 [33.1, 40.5]	36.7 (9.8) 35.0 [35.1, 38.4]	0.67	0.381	35.9 (6.7) 36.0 [33.8, 38]
Fusion of the spine								
Number of fused vertebrae	-	-	-	10.6 (1.6) 10.0 [10.2, 11]	9.5 (1.5) 9.0 [9.25, 9.75]	< 0.001	-	-
Fusion from T4 or above	-	-	-	30 (65%)	48 (36%)	0.001	-	-
Fusion to L1 or above	-	-	-	20 (43%)	38 (28%)	0.054	-	-
At the present follow-up								
Trunk deformity, degrees	10.5 (4.2) 10.0 [9.46, 11.5]	9.9 (5.5) 10.0 [8.83, 11]	0.438	16.2 (6.7) *** 17.0 [14.4, 18]	10.9 (5.6) 10.0 [9.96, 11.8]	< 0.001	< 0.001	9.0 (4.1) 9.5 [7.73, 10.3]
Oswestry Disability index a)	7.8 (8.9) 4.0 [5.6, 10]	7.6 (9.0) 4.0 [5.85, 9.35]	0.567	10.3 (13.0) 6.0 [6.8, 13.8]	8.4 (10.0) 4.0 [6.71, 10.1]	0.443	0.478	7.7 (8.3) 5.0 [5.13, 10.3]
Physical strain at work								
Levels 1–2	44 (70)	65 (64)	0.420	35 (66)	86 (64)	0.764	0.662	27 (67)
Levels 3–4	19 (30)	37 (36)		18 (34)	49 (36)			13 (33)
Physical strain during leisure time								
Levels 1–2	35 (56)	72 (71)	0.049	38 (72)	112 (83)	0.084	0.073	23 (57)
Levels 3–4	28 (44)	30 (29)		15 (28)	23 (17)			17 (43)

P-value between AIS untreated and the other groups of patients is presented as * = *p* ≤ 0.05, **p ≤ 0.01, *** = *p* ≤ 0.001

**Table 5:** Spinal mobility and muscle endurance in patients who were brace treated, surgically treated or untreated. Mean (SD), median [CI 95%: lower, upper]

	Brace-treated patients			Surgically treated patients				Untreated patients
	Onset until age 10 n = 63	Adolescent onset n = 102	p-value	Onset until age 10 n = 53	Adolescent onset n = 135	p-value	p-value Onset until age 10 BT vs ST	Adolescent onset n = 40
Spinal range of motion								
Thoracic mobility								
Thoracic rotation to the right (degrees)	41.5 (9.3) 40 [39.2, 43.8]	38.7 (7.7) ** 40 [37.2, 40.2]	0.098	37.4 (10.3) ** 40 [34.6, 40.2]	28.8 (8.6) *** 30 [27.4, 30.3]	< 0.001	0.068	44.5 (12.7) 45 [40.6, 48.4]
Thoracic rotation to the left (degrees)	40.1 (10.0) * 40 [37.6, 42.6]	36.3 (8.0) *** 40 [34.8, 37.8]	0.015	33.6 (7.7) *** 35 [31.5, 35.7]	28.4 (8.8) *** 30 [26.9, 29.9]	< 0.001	< 0.001	44.4 (13.0) 45 [40.4, 48.4]
Thoracic total range of motion, flexion + extension (degrees)	40.1 (8.8) * 39 [37.9, 42.3]	28.4 (10.9) * 29 [26.3, 30.5]	< 0.001	15.2 (8.3) *** 15 [13, 17.4]	15.5 (9.6) *** 13 [13.9, 17.1]	0.390	< 0.001	34.2 (13.5) 35 [30, 38.4]
Lumbar mobility								
Lumbar total range of motion, flexion + extension (degrees)	77.9 (17.7) *** 79 [73.5, 82.3]	54.7 (23.6) *** 59.5 [50.1, 59.3]	< 0.001	55.2 (16.2) *** 54 [50.8, 59.6]	33.9 (17.3) *** 36 [31, 36.8]	< 0.001	< 0.001	87.7 (13.1) 88.5 [83.6,91.8]
Lumbar forward flexion (cm)	5.4 (1.3) *** 5.5 [5.08, 5.72]	5.4 (1.1) *** 5.5 [5.19, 5.61]	0.807	4.4 (1.3) *** 4.2 [4.05, 4.75]	3.6 (1.5) *** 3.5 [3.35, 3.85]	0.003	< 0.001	6.3 (1.0) 6 [5.99, 6.61]
Mobility of the full spine								
Backward bending of the whole spine (cm)	5.2 (2.6) ** 5 [4.56, 5.84]	3.3 (1.6) *** 3.0 [2.99, 3.61]	< 0.001	2.8 (1.3) *** 2.7 [2.45, 3.15]	1.5 (1.14) *** 1.5 [1.31, 1.69]	< 0.001	< 0.001	6.7 (2.8) 6 [5.83, 7.57]
Finger-floor distance (cm)	6.6 (9.8) 0.00 [4.18, 9.02]	7.0 (9.6) 0.00 [5.14, 8.86]	0.702	8.8 (10.8) * 4.0 [5.89, 11.7]	12.2 (12.2) *** 10.0 [10.1,14.3]	0.092	0.389	4.2 (6.6) 0.0 [2.15, 6.25]
Muscle endurance								
Endurance of trunk flexors (sec)	140.0 (82.2) 121.5 [120, 160]	106.1 (78.8) *** 87 [90.8, 121]	0.002	125.4 (91.8) * 98.5 [101, 150]	104.7 (85.9) *** 76.5 [90.2, 119]	0.130	0.113	158.3 (85.4) 144 [132, 185]
Endurance of trunk extensors (sec)	255.5 (80.6) 300 [236, 275]	169.8 (97.0) *** 170.5 [151, 189]	< 0.001	234.7 (90.4) 300 [210, 259]	138.6 (106.5) *** 108.0 [121, 157]	< 0.001	0.300	234.0 (94.2) 300 [205, 263]
Subjective feeling of back stiffness								
VAS (0–100)	30.2 (27.5) 26.0 [23.1, 37.1]	30.4 (27.8) 20.0 [24.9, 35.9]	0.687	32.0 (28.2) 26.0 [24.2, 39.8]	32.6 (28.2) 23.0 [27.8, 37.4]	0.718	0.634	-

ROM: Range of motion. P-value between AIS untreated and the other groups of patients is presented as * = *p* ≤ 0.05, ** = *p* ≤ 0.01, *** = *p* ≤ 0.001

The thoracic total ROM of the brace treated EOS group (mean 40.1°) was significantly larger than in both the untreated AIS patients (34.2°, *p* < 0.05) and the braced AIS patients (28.4°, *p* < 0.05). The lumbar total ROM of the BT EOS group (mean 77.9°) was 9.8° less than among the untreated patients but significantly better than among the braced AIS patients (54.7°).

The thoracic total mobility of the operated EOS patients was similar to that of the operated AIS patients, but the lumbar total ROM was significantly less in the operated AIS patients (33.9° vs. 55.2°). The lumbar ROM was, therefore, evaluated according to the lowest level of the fusion. Both EOS groups, with fusion down to L1/above or to L2/below, had significantly better ROM than equivalent AIS patients (for fusion to L1/above, 61.2° vs. 40.1° and for fusion to L2/below 51.6° vs. 31.4°, respectively, *p* < 0.001).

### Muscle endurance of trunk flexors and extensors

Muscle strength (measured by endurance of trunk flexors and extensors) did not differ significantly between the braced and operated EOS groups, Table 5.

Both the trunk flexor and extensor strengths in the braced EOS group were similar to that of the untreated, and younger, AIS group. The braced AIS group had significantly less strength of flexors and extensors compared to the untreated AIS group and the braced EOS group (braced AIS vs. braced EOS: 106.1 vs. 140 s, *p* = 0.002 and 169.8 vs. 255.5 s, *p* < 0.001).

The trunk extensor strength of the operated EOS patients was similar to that of the untreated AIS patients, while the operated AIS group had significantly less strength (138.6 s.) compared to both these two groups (234.7 EOS and 234.0 untreated AIS, *p* ≤ 0.001 for both).

### Back pain and function

The majority of the EOS patients (86%) reported a normal back function (score ≤ 20) as measured by the ODI. Six brace treated and four surgically treated patients reported a score between 21 and 40 representing moderate disability and two ST patients reported a score between 41 and 60 representing severe disability. There were no significant differences compared to the BT or ST AIS patients.

The level of physical strain during work and leisure time showed no significant differences between the EOS groups.

The subjective feeling of back stiffness showed no significant differences between the early onset groups or compared to the BT or ST AIS patients (Table 5). There was no correlation between the subjective feeling of back stiffness and the length of the fusion in the ST EOS group.

### Correlations between ROM and trunk muscle endurance vs back function

For operated patients of the EOS group, the Oswestry Questionnaire showed a low correlation (rs = 0.49, *p* < 0.001) towards the lumbar ROM as well as with the subjective feeling of back stiffness (rs = 0.41, *p* < 0.01). Only six operated patients had ODI > 20, and these six all had a lumbar range of motion below the mean value. Correlation between the subjective feeling of back stiffness and lumbar ROM was also found in the braced EOS group (rs = 0.33, *p* = 0.007).

The physical activity during leisure time correlated with trunk flexor endurance in the BT EOS group (rs = 0.32, *p* < 0.01), and the ST EOS group (rs = 0.32, *p* = 0.01). No other correlations could be found towards ROM and endurance tests.

## Discussion

One important finding in this long-term follow-up is that patients brace treated for EOS have similar ROM and TME as untreated patients with AIS and significantly better than brace treated AIS patients. This was seen although they were significantly younger at start of treatment and bracing time was significantly longer than in the AIS patients, a fact that one could assume would instead lead to the opposite outcome. These findings indicate that bracing started at an early age does not negatively affect muscle endurance or thoracic ROM compared to untreated patients. The majority, 92%, of the EOS group was diagnosed between the age of four and ten years, which reflects a more juvenile onset than an onset at a very young age. This explains the treatment start after age ten and reflects that the group with onset before age ten in fact were not so different to the adolescent onset group. The age at start of treatment in the adolescent group is fairly late, reflecting the fact that many patients came late for treatment during the 1970’s, when bracing for scoliosis was started.

The reason for less reduced ROM and TME among those with somewhat earlier onset and longer bracing time is problematic to explain. No other publications had been found to compare our results with. The treatment was equal in both groups, the same type of brace was given and patients were equally instructed and encouraged to be physically active regardless of age. One may speculate if this difference in spinal range of motion depends on the earlier start of brace treatment. Children at a younger age need less pressure from the brace to get a good “in-brace-correction” and they also get used to the brace easier, and feel less limited by the brace and will therefore continue their natural body movements. Conversely, the older the child is at the start of bracing, the more pressure is needed within the brace to get a good correction and the more restricted the child will feel, leading to less activity and less body movements. Furthermore, the older child has an increased understanding of what the back problem and treatment entails. In some children this might lead to insecurity and perhaps even fear, which contributes to caution and activity limitations.

The second important finding was that the ST patients with EOS were neither weaker nor stiffer compared to ST patients with a later onset scoliosis, in contrast to our assumptions. In fact, the reduction of the ROM was lower among the surgically treated EOS patients than among the AIS patients, despite somewhat longer fusions in the EOS group. This was seen even when analyzing subgroups with similar caudal end of fusion in the lumbar region. For endurance of trunk extensors, no reduction at all was seen for the whole EOS group compared to the untreated AIS patients. A possible explanation for the difference towards that AIS patients might be that more patients with AIS were surgically treated during the period when long postoperative brace wear was routine at the treating department, which possibly might contribute to this outcome. As we have not been able to find any other publications presenting results for similar groups, comparisons cannot be made.

Although weak, correlations were found between lumbar ROM versus Oswestry Disability Index for the operated and versus subjective feeling of back stiffness for both the braced and operated EOS patients. Trunk flexor endurance and physical activity during leisure time showed weak correlations in both of the EOS groups. Muscle endurance has previously been shown to affect back function in operated AIS patients [14, 35] and it was also found that the braced AIS patients estimated their backs to be stiffer than controls [36].

Reliability and validity are important factors when evaluation of results of outcome measures in clinical studies. Some of our variables have been shown to have excellent clinimetrics, as the Schober test; however, the coefficient of variation in the measurements with the Debrunner kyphometer has been studied and found to be 8.6% for the thoracic ROM and 4.4% for the lumbar ROM [28]. The significant differences of the measurements of the thoracic and lumbar ROM in the present study varies between 9.8° and 32.5°. The clinical value of the reduction is not established but, due to the size of existing differences, could be considered important. Concerning TME, the tests used in this study have previously been shown to have a high reliability and reproducibility [30]. The significant differences between performed measurements of the TME vary between 14.6 and 96.1 s in the present study. The clinical value of these reductions may also be considered to be important.

A weakness in the assessment of the results of the present study is that all the treated patients were around 40 years of age at the present follow-up, while the untreated patients used for comparisons were significantly younger, around 32 years. Previously published studies, Yukawa et al. [37] and a meta-analysis by Arshad et al. [38], have reported significantly decreased ROM with increasing age. For the age groups of interest in this study, 30 and 40 years of age, the reduction of total lumbar ROM was only 7% for male and 8% for women in the first study [37], while the second study [38] found reductions of range of extension and of flexion by approximately 22% in males and six and two per cent, respectively, in females.

Significant reductions were found for lumbar range of motion in both braced groups, with ten degrees in the early and around thirty degrees in the adolescent onset patients. After recalculation of our values and comparing with the untreated AIS group, the lumbar ROM was 11,2% lower in the braced EOS group, which consisted of 86% females, meaning that only a few per cent of the reduction is not age related compared to the published data of 8% age related changes [37, 38]. We were surprised by the small reduction of lumbar ROM in this early onset patient group, despite the fact that many individuals had undergone full-time bracing for many years.

The braced AIS group on the other hand, with 96% females, had a lumbar range of motion that was 37.6% lower than the untreated AIS patients, which is far more than the reduction of about 8% expected by aging. One theory for this finding, discussed above, might be an easier adaption to brace treatment at a younger age.

Sasaki et al. [39] have examined healthy individuals between 18 and 89 years of age and found trunk extension strength to be higher for extension than for flexion, which was also found in this study. They noted a significantly decreased trunk muscle strength after the age of sixty, earlier for flexion than for extension strength [39]. This might partly be caused by the reduction of the cross-sectional area of the paraspinal muscles, which decrease with age [40]. We found clear reductions of trunk strength in all study groups towards the untreated AIS patients, but as all groups were well below age sixty, no differences due to age could be expected. The differences between those with earlier and adolescent onset cannot be explained by treatment methods, it was performed with the same indications and methods and during the same time period. The fact that we were unable to find other published studies of previously treated patients for comparison points out the need for further studies in this area.

One strength of this study is the long follow-up time of the group of 116 patients with EOS, treated with brace or surgery before skeletal maturity. To our knowledge, the spinal range of motion, trunk muscle endurance and lumbar spine stability has previously not been evaluated. A limitation of the study is that we were not able to compare the results to a group of healthy controls, instead we used results from an untreated group of AIS patients.

## Conclusion

At a mean of 26.5 years after completed treatment due to early onset idiopathic scoliosis, it was found that:

1. Our group of braced EOS patients, with mainly but not only juvenile onset, have similar range of motion and trunk muscle endurance as untreated, somewhat younger, patients with AIS. They were also significantly more mobile than braced AIS patients despite a longer treatment time in brace.
2. The endurance of trunk extensors was not reduced in surgically treated EOS patients compared to the untreated AIS patients.
3. The operated EOS patients were neither weaker nor stiffer compared to operated AIS patients, despite somewhat longer fusions in the EOS group.
4. The degree of lumbar range of motion was found to affect back function in operated EOS patients.
5. Endurance of trunk flexors correlated with physical activity level during leisure time.
